# Developing cardiovascular disease risk programs in India—Why location and wealth matter

**DOI:** 10.1371/journal.pmed.1002582

**Published:** 2018-06-19

**Authors:** David Peiris, Dorairaj Prabhakaran

**Affiliations:** 1 The George Institute for Global Health, UNSW Sydney, Sydney, Australia; 2 Centre for Chronic Disease Control, New Delhi, India; 3 Public Health Foundation of India, New Delhi, India

## Abstract

In a Perspective, David Peiris and Dorairaj Prabhakaran discuss implications and challenges of cardiovascular disease risk assessments in the population of India.

## CVD risk stratification for population health

Assessment of a person’s total or absolute risk of a cardiovascular disease (CVD) event based on multiple risk factors is superior to assessment of single risk factors when identifying who is at greatest risk of a CVD event [1]. In this issue of *PLOS Medicine*, a study by Rifat Atun and colleagues outlines the use of CVD risk prediction equations for population health rather than for clinical purposes [2]. This has inherent appeal because risk stratification of a population could be used to drive policy and health service planning, inform funding decisions around subsidising treatments, and identify particular subgroups for more intensive intervention strategies.

Atun and colleagues used 2012 and 2014 household health survey data to assess sociodemographic and geographic variation in CVD risk profiles across most states and union territories in India. Cross-sectional estimates of 10-year CVD risk were derived using the Framingham risk equation (FRE) for people aged 30 to 74 years, calibrated for India using 2015 Global Burden of Disease data. Sensitivity analyses were conducted using three other risk prediction equations that have also been calibrated with Indian data. The main finding is the identification of regional ‘hotspots’ of relatively higher CVD risk scores regardless of which risk prediction equation was used. Furthermore, positive associations were found with increasing household and district wealth in both rural and urban areas. Although urban areas overall had a stronger positive association with higher CVD risk scores, the district wealth gradient was more pronounced in rural compared with urban areas, and the household wealth gradient was greater for females compared with males.

The multivariable, absolute risk paradigm is now commonplace in many management guidelines around the world; however, there remain challenges with its implementation. First, the validity of cohort-derived risk prediction equations applied to other populations is frequently questioned [3]. Second, when risk factor prevalence rates and CVD incidence rates are rapidly changing due to demographic and socioeconomic changes in the community, the need to recalibrate such equations becomes important [4]. Third, when assessing the effectiveness of risk-based interventions, most studies are based on simulation studies [5] rather than empirical trial data [6]. Finally, although risk prediction equations were designed to guide decisions on management and intervention, the evidence that use of risk scores improves clinical outcomes remains unclear [7].

The limitations of Atun and colleagues’ study are related to these broader challenges of implementing the risk-based paradigm. First, none of the equations used have been validated with longitudinal data from Indian cohorts. Consequently, the absolute values of the risk profiles presented in this paper cannot be relied on. The wide variation in risk estimates between the FRE scores and WHO risk charts and Globorisk scores is concerning, and there is currently no way of knowing which is the most accurate. Second, the survey data that were relied on are now over 5 years old, and, given rapid demographic transitions across India, the risk estimates may not reflect the contemporary situation. This is a general limitation with risk prediction equations. WHO risk charts are currently being recalibrated with country-specific data, but this is the first time this has been done since the original risk charts were published in 2007. Third, 27% of the survey sample did not have sufficient information to assess risk, and males with missing data appear to be a little better educated, wealthier, and more likely to reside in an urban setting. Finally, the surveys did not ascertain a history of a prior CVD event. Assuming that the entire population studied are event free will greatly underestimate risk profiles.

### Implications for cardiovascular risk reduction in India

The implications of this work are complex and inevitably will mean different things for different sectors. Epidemiologists may be unimpressed by the variation in risk profiles generated by the four equations and conclude that, in the absence of a gold standard equation, the principal findings are of questionable value. Clinicians and health service administrators may be focussed on the challenges of implementing absolute risk assessments in clinical practice. India, like many countries, uses hybrid guidelines that include a combination of risk scores and single risk factor thresholds for determining treatment decisions. For example, those with an elevated blood pressure of greater than or equal to 160/100 mmHg are considered to be clinically high-risk regardless of their risk scores [8]. This approach seeks to strike a middle ground for those who may have reservations about a pure risk-based approach and may make it easier for nonphysician workers when conducting household screening assessments [9]. These implementation challenges highlight the need for reliable equations with variables that can be easily and cheaply measured by staff with low levels of training. An important implication from Atun and colleagues’ study is that cohort derived-equations in India that incorporate measures of wealth and geographic location, such as those derived in the United Kingdom and Scotland, may be needed [10].

For policy makers, however, it would be unwise to wait for perfect data to create the perfect policies. Access gaps to essential care are large, and action is needed now. There are broadly two main policy levers to lower CVD risk—population- and individual-level approaches. Population-level approaches attempt to shift the distribution of risk of the whole community through policies, laws, and regulations (e.g., improved food supply, healthy environments, tobacco control). The risk profile heat maps generated in Atun and colleagues’ study could guide where the most intensive efforts are needed. Individual-level approaches consist of CVD risk assessment programs implemented in healthcare facilities with thresholds set for defining intervention intensity. A universal screening program for all adults is unlikely to be the best use of scarce resources, and targeted programs are likely to be more cost-effective [11]. A more detailed appraisal of regional risk profiles, such as those generated in Atun and colleagues’ study, could therefore assist with economic modelling and investment decisions.

Furthermore, given the wide variation in risk profile, the resource implications for intervention similarly will vary greatly by region. Health technology assessments could play a vital role in driving these decisions. India’s new Medical Technology Assessment Board will engage multisectoral representatives to provide recommendations on the value of particular health interventions, programs, and technologies [12]. These assessments could better inform strategic purchasing arrangements and the design of benefit packages that are based on population need rather than simply trying to meet service demand [13].

Although better risk profile data are critical for the design and funding of evidence-based programs, an equally important challenge is overcoming implementation barriers of these programs. The National Programme For Prevention and Control of Cancer, Diabetes, Cardiovascular Disease and Stroke, established in 2008, has faced major resource challenges to build primary healthcare workforce capacity and address both supply and demand side barriers to implementation [14]. The recently announced Ayushman Bharat National Health Protection Scheme plans to provide publicly funded health insurance to cover over 100 million poor families in India and upgrade over 150,000 subcentres to health and wellness centres [15]. Although there are major questions regarding the resourcing and management of such an ambitious program, it does provide the opportunity for revitalisation of the primary healthcare system, new workforce initiatives, and improved access to affordable treatments. Data-informed, regionally specific CVD risk programs could play a central role in such programs and drive system improvements across India.

## Abbreviations

CVD: cardiovascular disease
FRE: Framingham risk equation
